# Bacterially Delivered miRNA-Mediated Toll-like Receptor 8 Gene Silencing for Combined Therapy in a Murine Model of Atopic Dermatitis: Therapeutic Effect of miRTLR8 in AD

**DOI:** 10.3390/microorganisms9081715

**Published:** 2021-08-12

**Authors:** Wonsuck Yoon, Eun-Jae Kim, Yongsung Park, Seunghyun Kim, Yong-Keun Park, Young Yoo

**Affiliations:** 1 Allergy Immunology Center, Korea University, Seoul 02841, Korea; dcomtrue@korea.ac.kr (Y.P.); seunghyun@korea.ac.kr (S.K.); 2 Department of Life Science and Biotechnology, Korea University, Seoul 02841, Korea; wonsucky@gmail.com (E.-J.K.); actsbio@naver.com (Y.-K.P.); 3 Department of Pediatrics, Korea University Medical College, Seoul 02841, Korea

**Keywords:** TLR8, atopic dermatitis, mouse model, *Salmonella typhimurium*

## Abstract

In atopic dermatitis (AD), skin inflammation is caused by complex interactions between genetic disposition and aberrant innate/adaptive immune responses. Toll-like receptors (TLRs) are key molecules in the innate/adaptive immune response as they recognize various molecular motifs associated with pathogens. Among them, TLR8 is implicated in eczematous skin reactions. We investigated the combined therapeutic effects of TLR8 gene silencing by the bacterial delivery of miRNA. We used *Salmonella* as a vector to deliver TLR8 miRNA. The recombinant strain of *Salmonella enterica* subsp. *enterica* serovar Typhimurium (ST) expressing TLR8 miRNA (ST-miRTLR8) was prepared for knockdown of TLR8. After oral administration of ST-miRTLR8 into mice, we observed the cytokine levels, skin pathology and scratching behaviors in an AD-like mouse model. TLR8 down-regulation decreased macrophage-derived chemokine concentrations in activated human mast cells. Serum IgE and interleukin-4 production were suppressed whereas IFN-γ was induced after oral administration of ST-miRTLR8. Scratching behaviors and skin inflammation were also improved. In addition, attenuated *S. typhimurium* safely accumulated in mouse macrophages and showed adjuvant effects. This study shows that the recombinant miRNA that expresses the TLR8 miRNA has therapeutic effects by suppressing Th2 inflammation. TLR gene modulation using miRNA via *Salmonella* vectors will thus have a double-protective effect in the treatment of AD.

## 1. Introduction

Atopic dermatitis (AD) is a chronic condition that usually begins in childhood but often persists into adulthood [1]. Although the exact etiology is unclear, it is clear that the complex interplay between barrier dysfunction, inflammation and pruritus is important for development, progression and chronicity. Skin inflammation is related to abnormalities of filaggrin, intercellular lipids, tight junctions, thymic interstitial lymphoetin and toll-like receptors (TLRs) [2,3]. Skin inflammation comprises complex interactions between genetic predisposition, imbalanced systemic and/or local immune responses and skin barrier dysfunction [4]. In AD, variations in the innate immune responses can prime a deviation of the adaptive immune response, skewing to dominance of Th2 cytokines such as interleukins (ILs) 4, 5 and 10 [5].

The pathogenesis of AD is multifactorial and both genetic and environmental disposition is crucial. In addition, exposure to microbial pathogens is associated with the development of AD. TLRs are key molecules in the innate immune response as well as antigen-specific adaptive immune mechanisms as they recognize various molecular motifs associated with pathogens or tissue injury [6]. Following exposure to micro-organisms, TLRs are also triggered, resulting in cytokine release and inflammation. Activation of different TLRs promotes Th1 cell differentiation and thereby limits Th2 cell development. They have been implicated in inflammatory skin diseases including AD [7]. In the previous study with severe adult patients with AD, disturbance of the expression and function of TLR showed an association with protective inflammation and either led to bacterial colonization or hampered the Th1 response/Th2 shift [8].

Among the TLRs, TLR8 recognizes single-stranded RNA (ssRNA) and induces NF-kB via MyD88 signaling, which can produce pro-inflammatory cytokine genes, such as IL-1, IL-6, IL-12 and TNF-α, from peripheral blood mononuclear cells (PBMCs), monocytes and dendritic cells (DCs) [9]. Decreased IFN-γ and TNF under TLR7/8 stimulation in DCs were observed in patients with AD [8]. TLR8 is a key component of the innate immune system; activation of TLR8 signaling plays a significant role in the pathogenesis of AD [10,11]. Therefore, we chose the TLR8 gene as a therapeutic modality for reducing the inflammatory response in AD.

Meanwhile, microRNAs (miRNAs) are expressed as ssRNA sequences that are 22 nucleotides in length and naturally direct gene silencing through components [12]. Although it is known that TLR activation induces miRNAs, which may participate in various mechanisms to control excessive inflammation [13], functional data showing the exact effects of miRNAs on TLR responses are still limited. Thus, it is necessary for us to reveal the functional consequence of miRNA expression and the mechanisms through which it affects innate immunity. 

In genomics, miRNA has been considered an extremely useful experimental tool to suppress the gene. However, despite the great potential of miRNA, clinical trials with it are difficult because of its transient nature, instability and the lack of efficient delivery methods to the target site. When orally delivered to a mouse, *S. typhimurium* strains engineered to express IL-12 or granulocyte/macrophage colony-stimulating factors mediate cytokine gene expression and exert genetic effects [14]. Furthermore, oral administration of attenuated *S. typhimurium* itself has been shown to restore the production of IFN-γ in macrophages of IFN-γ-deficient mice [14]. Therefore, it is plausible that *Salmonella* can be used to deliver expression vectors encoding various effector genes to cells, with the aim of enhancing endogenous genetic modulation. 

This study investigated the efficacy of live, attenuated *S. typhimurium* as a carrier for oral gene delivery therapy, and the potential of TLR8 miRNA for the modulation of the inflammatory response in an AD-like mouse model.

## 2. Materials and Methods

### 2.1. Construction of miRNA Expression Vectors

Two single-stranded DNA oligonucleotides were designed, encoding the TLR8 target pre-miRNA (GenBank accession no. NM_133212) by the RNAi design program (Invitrogen, Carlsbad, CA, USA). Top and bottom single-strand oligos (top strand: 5′-TGCTGAAACCAGGTAGAAGGAATCGTGTTTTGGCCACTGACTGACACGATTCCCTACCTGGTTT-3′, bottom strand: 5′-CCTGAAACCAGGTAGGGAATCGTGTCAGTCAGTGGCCAAAACACGATTCCTTCTACCTGGTTT-3′) were annealed to generate a double-strand oligonucleotide for cloning into the miRNA expression vector. Oligonucleotides encoding miRNA against mouse TLR8 genes were inserted into the pcDNA™ 6.2-GW/EmGFP-miR expression vector (Invitrogen), generating the miRTLR8 plasmid. The scrambled miRNA-expressing plasmid was named miR*CV*. The plasmid-generated miRNA was detected using a green fluorescent protein (GFP) by Western blot analysis and fluorescence microscopy. 

### 2.2. TLR8 miRNA-Expressed Salmonella Strain

The resultant plasmid vectors were transformed into competent *Escherichia coli* DH5α. The plasmid DNA was isolated from the DH5α cells using a plasmid mini-prep kit (GeneAll, Seoul, Korea). This plasmid DNA was transformed into the *S. typhimurium* SF586 strain (SF586) by electroporation. Then, these plasmids from the transformed SF586 were transformed into attenuated *S. typhimurium* BRD509.

### 2.3. Western Blot Analysis

The whole cell of bacterial culture was run on am SDS-PAGE and electrophoretically transferred to nitrocellulose membranes. The membranes were pre-equilibrated with TBS-T solution containing 5% skim milk overnight and were incubated with a mouse anti-GFP antibody. The membranes were incubated with a goat anti-mouse IgG HRP conjugate at room temperature. Immune reactive protein bands were visualized using chemiluminescence blotting substrate.

### 2.4. Invasion Assay

The murine macrophage cell line (RAW 264.7 cells) was cultured in Dulbecco’s modified Eagle’s medium (DMEM) with 10% fetal bovine serum (FBS) (Welgene Inc., Gyoungsan-si, Korea) supplemented with antibiotics (100 units/mL penicillin, 100 mg/mL streptomycin; Sigma-Aldrich CO., St. Louis, MO, USA). RAW 264.7 cells were allowed to grow in the wells overnight, creating a flat layer. *Salmonella* was separately grown overnight. On the next day, the RAW 264.7 cells were inoculated with the *Salmonella* at a MOI of 100:1 and were incubated together for 1 h. Centrifuging the plates for a few minutes may help bring cells and *Salmonella* in contact and initiate infection. After that, the cells were washed three times with PBS and then treated with 100 µg/mL gentamycin (Invitrogen, Carlsbad, CA, USA) solution for 1 h to kill *S. typhimurium* remaining outside the cells. The plates were then washed well to remove the dead bacteria. After that, RAW 264.7 cells were lysed using 1% Triton X-100 for 5 min at 37 °C to detect *Salmonella*. They were incubated on LB plates by plating 10-fold serial dilutions. The colony-forming units (CFUs) were counted the next day.

### 2.5. Reverse Transcription PCR

For reverse transcription PCR (RT-PCR) analysis at 8 h after transfection, cells were collected and total RNA was quantified using the TRIzol reagent (Invitrogen, Carlsbad, CA, USA): IL-1α, IL-1β, IL-18, CCL17, CCL22 and TLR8. 

### 2.6. AD-Like Mouse Model

Four-week-old CD-1 (ICR) female mice were housed in an animal room maintained at 24 ± 2 °C, with a 12 h light/dark cycle. A 2,-4-dinitro-1-chlorobenzene (DNCB) (Sigma-Aldrich, St. Louis, MO, USA) solution was used as a contact allergen. Mice were sensitized with 100 μL of 1% DNCB (olive oil: acetone, 1:3) for three days. After 48 h, the mice were treated with 200 μL of 1.5% DNCB daily for three days. Elicited scratching behaviors were measured. All mouse experiments were performed in accordance with the institutional animal protocols and guidelines set by Korea University.

### 2.7. Cytokine Analysis and Bacterial Distribution in an AD-Like Mouse Model

Another set of mice were sacrificed to excise each tissue for bacterial distribution. The other diluted tissue homogenates were plated onto LB agar containing spectinomycin in duplicate, and the colony count was determined the next day. An AD-like mouse model was orally inoculated with 1.6 × 10^8^ *S. typhimurium* (ST)-miRTLR8, ST-miRCV and PBS. One week after inoculation, one set of mice was sacrificed to excise the spleen and the skin. Tissue samples were used for cytokine RT-PCR. 

### 2.8. Enzyme-Linked Immunosorbent Assay (ELISA)

Serum samples were obtained from the control and test groups by eye bleeding from mice one-week post-recombinant bacteria treatment. The total sera were used for measurement of IgE and IL-4 levels by ELISA assay and written informed consent for experimental use of the serum specimens was obtained from all patients. The study was approved by the local ethics committee of Korea University Hospital.

### 2.9. Observation of Scratching Behaviors

Mice were placed in an acrylic cage and the frequency of upper-back scratching by the hind paws was counted. Scratches were scored over a 10-min period and the mice were observed for seven days. 

### 2.10. Skin Histology

Portions of the dorsal skin were fixed with 10% neutral formalin, embedded in paraffin and sectioned at 4 μm thickness. Sections were stained with hematoxylin-eosin.

### 2.11. Flow Cytometry

Splenic cells obtained directly from tissues were resolved to a single-cell suspension by a nylon mesh and we removed red cells with a lysis buffer. The rest of the single-cell suspension was stimulated with phorbol myristate acetate (PMA) plus ionomycin. These cells were then stained, fixed and permeabilized using FACS perm solution according to the manufacturer’s instructions. Th17 cells were analyzed by the Th17 FACS kit.

## 3. Results

### 3.1. TLR8 Down-Regulation Suppressed Inflammatory Responses in Activated Human Mast Cells

**Figure 1:**
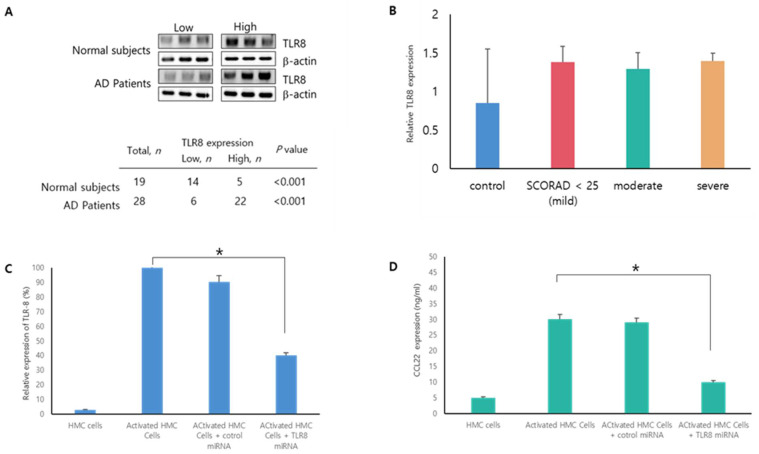
TLR8 expression in the peripheral blood mononuclear cells of patients with atopic dermatitis (AD) and TLR8 miRNA effects in activated HMC-1 cells. (**A**) TLR8 was highly expressed in patients’ PBMC cells compared to the normal subjects’ by Western blotting. (**B**) The relative expression of TLR8 was higher in patients with AD than normal subjects. (**C**) Suppression of TLR8 was detected in PMA/ionomycin-treated HMC-1 cells after they were transfected with human TLR8 miRNA for 72 h. (**D**) CCL22 protein expression by TLR8 miRNA was suppressed in PMA/ionomycin-treated HMC-1 cells by ELISA assay. * *p* < 0.05 for the TLR8 miRNA group versus the activated control group.

We examined TLR8 expression in the PBMCs of patients with AD. Immunoblot analysis showed that TLR8 protein expression was significantly higher in the PBMC cells of patients with AD than in those of normal controls (Figure 1A,B). It is, therefore, assumed that TLR8 may be a promising target for AD therapy. Thus, we investigated whether TLR8-specific miRNA may serve as a therapeutic candidate for AD. We effectively knocked down the expression of endogenous TLR8 using TLR8 miRNA in activated HMC cells (Figure 1C), whereas the cytotoxicity of miRNA was not shown (Supplementary Figure S1). Next, a macrophage-derived chemokine (CCL22) expression test was performed. The CCL22 concentration in the supernatants was significantly lower in the miRTLR8 group compared to the control miRNA group or activated HMC-1 cells (Figure 1D). 

### 3.2. Construction and Analysis of TLR8 miRNA Plasmid Expression Vectors in S. typhimurium

**Figure 2:**
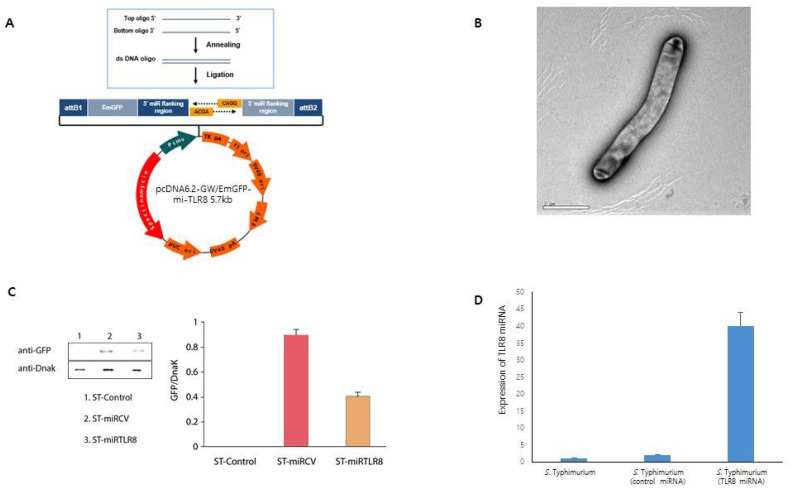
Construction of *Salmonella typhimurium* (*S. typhimurium*) expressing TLR8 miRNA. (**A**) Schematic representation of pcDNA6.2-GW/EmGFP-miR vector expressing TLR8 miRNA and co-cistronic reporter-gene Emerald green fluorescent protein (EmGFP). Both pre-TLR8 miRNA and EmGFP are co-expressed from a constitutive CMV promoter/enhancer. (**B**) The engineered *S. typhimurium* was visualized using electron microscopy. (**C**) To validate the production of genetically engineered *S. typhimurium* transformed with pcDNA6.2-GW/EmGFP-miRTLR8, the expression of co-cistronic GFP was measured. Cell lysates from genetically engineered *S. typhimurium* were resolved by electrophoresis, transferred to nitrocellulose membranes with an anti-GFP antibody. An anti-DnaK antibody was used as a control for the bacterial cytoplasmic protein. Lane 1: unmodified *S. typhimurium*, lane 2: *S. typhimurium* with pcDNA6.2-GW/EmGFP-miRNA negative control, lane 3: *S. typhimurium* with pcDNA6.2-GW/EmGFP-miRTLR8. GFP/DnaK expression was suppressed in ST-miRTLR8. (**D**) Also, miRNA was confirmed by real-time PCR in engineered *S. typhimurium* (unmodified *S. typhimurium* or genetically modified *S. typhimurium* expressing negative control miRNA or miRTLR8).

The main purpose of the present study was to investigate anti-inflammatory effects induced by TLR8 suppression and by attenuation of the *Salmonella* vector. To elicit their combined therapeutic effects, we cloned miRNA against TLR8 in *Salmonella*. The dsDNA oligo of TLR8 cloned into the pcDNA™ 6.2-GW/EmGFP-miR expression vector using T4 DNA ligase (Figure 2A). The recombined interfering plasmid was transformed into *S. typhimurium* (Figure 2B). To track the presence of miRNA in bacteria, we measured GFP expression (Figure 2C). As shown in Figure 2D, ST-miRTLR8 expressed TLR8 miRNA efficiently in culture lysate, indicating the successful construction of genetically modified *S. typhimurium* containing TLR8 miRNA expression vectors.

### 3.3. Effects of Bacterially Delivered TLR8 miRNA on Cytokine Levels in Activated Mast Cells

**Figure 3:**
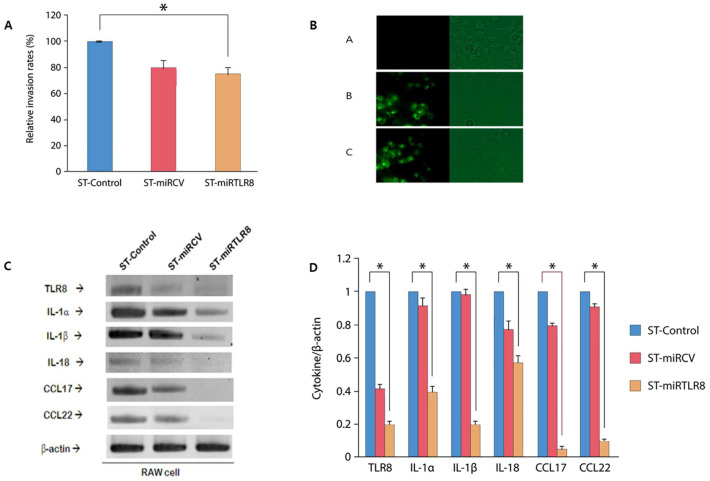
Invasion activity and modulation of cytokines/chemokines in genetically engineered *S. typhimurium* (ST). RAW mouse macrophages were infected with the indicated *S. typhimurium* at a multiplicity of infection (MOI) of 500 for 1 h (for invasion assay) or for 72 h (for cell viability and LDH assay). (**A**) Relative invasion rates were decreased in ST-miRTLR8 compared to the unmodified *S. typhimurium*-treated samples (* *p* < 0.05). (**B**) Green fluorescence was detected in the RAW 264.7 cells infected with either ST-miRTLR8 or ST-miRCV. (**C**) Green fluorescent protein (GFP) expression was detected in the RAW 264.7 cells in ST-miRTLR8 or ST-miRCV but not in the *Salmonella* control. Expression of cytokines (**C**) and quantification of cytokine levels (**D**)—such as TLR8, IL-1α, IL-1-β, IL-18, CCL17 and CCL22—were significantly decreased after treatment with ST-miRTLR8. * *p* < 0.05 for the ST-miRTLR8 group versus the ST-miRCV control group.

To determine the anti-inflammatory effects of ST-miRTLR8, we tested the invasion activity and cytokine modulation in RAW 264.7 cells with unmodified *S.* Typhimrium, ST-miRCV and ST-miRTLR8. To examine the expression of the miRNA vector and ST-miRTLR8, RAW 264.7 cells were infected with ST-miRTLR8, ST-miRCV and *Salmonella* counterparts (Figure 3A). Green fluorescence was detected in the RAW 264.7 cells infected with ST-miRTLR8 or ST-miRCV but not in the *Salmonella* controls (Figure 3B, Supplementary Figure S2).

In addition, expression and quantification of cytokine mRNA using RT-PCR from three separate experiments and normalized to that of β-actin were observed. Expression of cytokine mRNA (Figure 3C) and quantification of cytokine levels, such as TLR8, IL-1α, IL-1-β, IL-18, CCL17 and CCL22 (Figure 3D), were significantly decreased after treatment with ST-miRTLR8. IL-4 and IFN-γ were not activated in the activated mast cells and could not be measured in this experiment (data not shown).

### 3.4. Bacterially Delivered TLR8 miRNA-Modulated Humoral Immune Responses in an AD-Like Mouse Model

**Figure 4:**
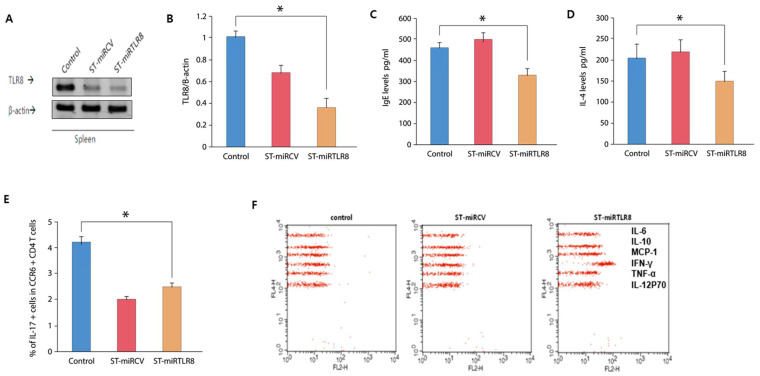
TLR8 suppression and changes in IgE, IL-4 and IL-17 levels in mice suffering from atopic dermatitis that were treated with ST-miRTLR8. Mice were orally infected with 1.6 × 108 CFU ST-miRTLR8 and ST-miRCV. One week after oral administration of ST-miRTLR8, suppression of TLR8 mRNA in the spleen (**A**) and quantification of cytokine mRNA levels (**B**) were observed. The serum levels of IgE (**C**) and IL-4 (**D**) were decreased by oral administration of ST-miRTLR8. There were slightly reduced Th17 cells in the ST-miRTLR8 treated mice compared to the controls. (**E**) In the blood, IFN-γ induction was increased above the other inflammatory cytokines (**F**) analyzed by cytometric bead array (CBA). * *p* < 0.05 for the ST-miRTLR8 group versus the ST-miRCV control group (*n* = 10).

To examine in vivo anti-inflammatory effects by ST-miRTLR8, an AD-like mouse model was orally inoculated with 1.6 × 10^8^ ST-miRTLR8, ST-miRCV and PBS. One week after oral administration of ST-miRTLR8, suppression of TLR8 mRNA in the spleen (Figure 4A) and quantification of cytokine mRNA levels were observed (Figure 4B). Furthermore, bacterial distribution without any histological inflammation was observed in the spleen, kidney and liver of the AD-like mice (Supplementary Figure S3).

The serum levels of IgE (Figure 4C) and IL-4 (Figure 4D) were decreased by ST-miRTLR8 oral administration. Figure 4E shows the significantly reduced number of Th17 cells in the ST-miRTLR8-treated mice compared to the control groups. Other inflammatory cytokines (IL-6, IL-10, MCP-1, IFN-γ, TNF-α and IL-12) were also analyzed by cytometric bead array (CBA) and showed that induction of IFN-γ in the blood was much increased compared to the other cytokines (Figure 4F). 

### 3.5. Bacterially Delivered TLR8 miRNA Suppressed Skin Inflammation in an AD-Like Mouse Model

**Figure 5:**
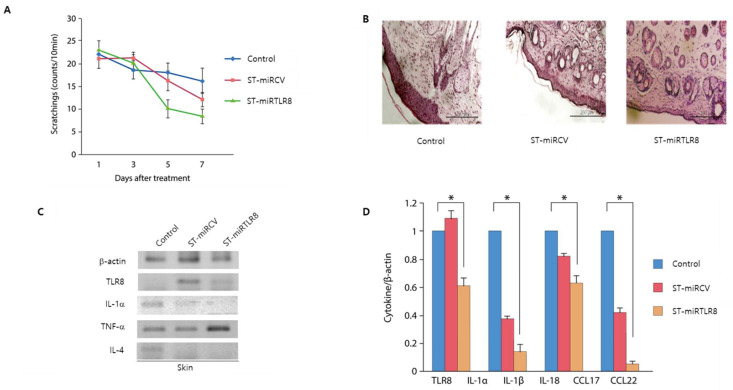
Decreased skin inflammation in an AD-like mouse model by oral administration of ST-miRTLR8 in mice. (**A**) The total scratch counts were dramatically decreased in the ST-miRTLR8-treated mice compared to the PBS or ST-miRCV groups. (**B**) Histological analyses were performed to examine the reduced inflammation due to ST-miRTLR8. (**C**) Immunoblot analysis showed suppression of IL-4 and IL-1a in the skin tissue from mice suffering from AD treated with ST-miRTLR8. (**D**) Cytokine levels were significantly decreased in the ST-miRTLR8 treated group. * *p* < 0.05 for the ST-miRTLR8 group versus the ST-miRCV-control group (*n* = 10).

To confirm the anti-inflammatory effects of ST-miRTLR8 in an AD-like mouse model, mice were placed in an acrylic cage and the frequency of upper-back scratching by the hind paws was counted. The scratching score was significantly decreased in ST-miRTLR8-treated mice compared to the PBS-treated mice (Figure 5A). The epidermal thickness and skin inflammation were decreased in the ST-miRTLR8-treated mice and the ST-miRCV group compared to the DNCB-treated control mice (Figure 5B, Supplementary Figure S4). Expression of IL-4 was considerably suppressed in the ST-miRTLR8–treated mice in RT-PCR (Figure 5C) as well as cytokine mRNA levels in the skin (Figure 5D). 

## 4. Discussion

The present study demonstrated that the suppression of TLR8 using the *Salmonella* vector decreased pro-inflammatory mediators in an AD-like mouse model. In particular, *S. typhimurium*-harboring plasmids which expressed TLR8 miRNA, reduced TLR8 gene expression in vitro and in vivo. We observed significantly decreased scratching behavior, skin inflammation and serum Th2 cytokine levels after oral administration of ST-miRTLR8 in an AD-like animal model. 

Our challenge in the future will be the development of more effective and safer drugs in the treatment of AD. However, given the complexity of immune pathways that lead to AD, more selective anti-inflammatory or immune-modulatory agents would be less effective. Thus, it is important to better characterize key immune pathways leading to the different phenotypes of AD because medications may vary in their effectiveness for the treatment of different phenotypes of AD. 

TLRs are key molecules in innate immunity that detect conserved structures, which exist in a broad range of pathogens, and either promote or inhibit inflammatory and immune responses. In our previous study, TLR8 was considered as one of the markers with significant changes in macrophages of the atopic dermatitis-like inflammatory response (data not shown). The role of TLRs in the pathophysiology of AD is not completely understood yet. However, recent studies found a specific polymorphism in TLRs 2, 5 and 9 in patients with atopic eczema [8,15,16]. Various immune cells, such as monocytes, macrophages, DCs, granulocytes and non-immune cells like keratinocytes, express TLRs and initiate a primary immune response. Once activated by TLRs, immune cells initiate phagocytosis/killing of pathogens and cytokine as well as chemokine production, leukocyte activation, and antigen presentation to T-cells, thereby initiating an adaptive immune response as well [6]. In order to clinically manipulate TLR, it should be altered through receptor antagonists, receptor agonists or single transduction inhibitors [17]. Currently, a TLR4 antagonist initially developed for sepsis therapy is being clinically tested for the treatment of allergic diseases [18,19].

In the present study, we intended to investigate how TLRs have been linked to atopic skin disease and their proposed therapeutic roles. In addition to complex interactions between defective epithelial barrier function, receptor expression, signaling pathways, and altered cytokine production, TLR-mediated activation or dysfunction has been attributed to development and exacerbation of AD. Thus, the manipulation of specific TLRs may lead to the development of novel therapies for allergic diseases. Because TLR expression is not confined to specific immune cells and has been detected in skin cells such as keratinocytes, it is not surprising that TLRs have been implicated in AD. The involvement of TLR8 in the etiology of AD was investigated by a recent clinical study [6]. That study revealed that production of anti-inflammatory cytokines, such as IL-10, IFN-γ and TNF, increased after TLR8 agonist stimuli in AD subjects compared to non-AD subjects [6]. TLR8 is not expressed on the cell surface but is found in endosomal compartments; its activation requires endocytosis to the pathogens. Intracellular TLR8 recognizes ssRNA and initiates downstream signaling events, leading to secretion of inflammatory cytokines that can influence the magnitude of the adaptive immune response. In the present study, we evaluated GFP expression in ST-miRTLR8 and ST-miRCV using fluorescent microscopy and Western blot analysis, observing significantly increased expressions in the ST-miRTLR8 and ST-miRCV bacteria groups, but not in the controls. 

Indeed, miRNA is expressed as small ssRNA sequences that are 22 nucleotides in length and naturally direct gene silencing through components shared with the RNAi pathway. It has been shown that miRNA expression profiles differ between disease and normal states. Previous studies on miRNA expression could determine functional and diagnostic roles of miRNA and now miRNA is recognized to modulate disease states by regulating RNAs at numerous levels [13,20]. In the present study, we used an RNAi mechanism to knock down TLR8, which has been considered a promising therapeutic approach to suppress disease-related gene expression. However, there have been problems with stability and delivery to target cells. Our previous research has shown that oral administration of miRCCL22 using *S. typhimurium* as a vector significantly reduced *CCL22* gene expression in the mouse spleen and thereby induced immune-modulatory effects in an AD-like mouse model [21]. Based on that research, we used *S. typhimurium* for invading and transferring the TLR8 miRNA expression vector into mucosal epithelial cells and showed that the attenuated *S. typhimurium* was able to deliver RNA expressing vectors targeting TLR8 and inducing RNAi in an AD-like mouse model. Other previous studies showed that *S. typhimurium* could transfer eukaryotic vector-based RNAi-expressing plasmids in vitro and in vivo by oral administration [11,12]. Therefore, it is pertinent to utilize *Salmonella* to deliver expression vectors encoding various effector genes to cells, with the aim of enhancing endogenous genetic modulation. 

Th2-associated skin inflammatory responses which are characterized by increased IL-4 and decreased IFN-γ secretion are implicated in the pathogenesis of the acute stage of AD [2]. We showed that administration of *S. typhimurium* expressing miRTLR8 reduced IL-4 and IgE levels and induced IFN-γ in DNCB-treated mice. Additionally, the IL-4, IL-1 and TNF-α levels were considerably reduced in the skin of mice inoculated with ST-miRTLR8. Because IL-4 is a key factor for inducing IgE isotype switching in B cells and IFN-γ inhibits IgE secretion through the antagonistic effect of Th2 cytokines in AD, imbalance of the Th1/Th2 immune responses is restored after oral administration of ST-miRTLR8.

In the present study, we used *S. typhimurium* as a vector to deliver TLR8 miRNA, leading to down-regulation of TLR8 genes and consequential decreases in Th2 cytokines. Also, we demonstrated that orally administered *S. typhimurium* safely accumulated in mouse macrophages. Our plasmid DNA, under the control of the CMV promoter, can be expressed in a eukaryotic system as well as a prokaryotic system. 

Macrophages are normal targets for *Salmonella* during natural infections. It has been demonstrated that attenuated bacteria can deliver nucleic acid vaccine constructs [22]. *Salmonella* makes for a reasonable tool for intestinal cell delivery because of its intracellular invasiveness. In particular, *Salmonella* strains, as biologics, produce and target microRNAs by themselves and can be more economically utilized in microbiome-mediated biologics research. We demonstrated that recombinant *Salmonella* could be used as an effective in vivo delivery system to transfer miRNA into immune cells such as murine macrophages. In addition, *Salmonella* itself contributes to adjuvant effects and induces Th1 cytokines and cell-mediated immunity [23,24]. A combination of *S. typhimurium* and miRNA may offer a more potent therapeutic modality in AD treatment than anti-TLR8miRNA alone, suggesting that TLR8 plays an important role in the restoration of Th2-skewed imbalance in AD and that *S. typhimurium* expressing TLR8 miRNA could potentially be used as an effective therapeutic modality for treating AD.

However, despite significant advances in the treatment of AD, the long-term therapeutic effects are not known well due to the multifarious causes of AD. Simultaneous silencing of other critical genes in AD is expected to achieve a more remarkable therapeutic effect. Our study demonstrated that modulation of miRNA may be implicated in AD therapy when a suitable miRNA delivery system is adopted to obtain the maximal therapeutic effects. Despite the conveniences and diversities of chemically synthesized miRNA, its clinical potential for systemic application was ultimately restricted due to a relatively short period of activity, the impractical dose of miRNA required and inaccurate delivery of therapeutic agents. Compared to synthetic miRNA, *S. typhimurium*-mediated vector-based miRNA may help achieve a higher targeting specificity and a more sustained miRNA effect.

In conclusion, the present study showed that the recombinant miRNAs that express the TLR8 gene had therapeutic effects by suppressing Th2 inflammation in AD. Furthermore, induction of Th1 cytokines and cell-mediated immunity by live attenuated *Salmonella* was observed. These results suggest that TLR8 gene modulation using miRNA via a *Salmonella* vector will have a double-protective effect against AD. Investigation of the modest regulation of TLR signaling by miRNAs and the synergistic effect with the *Salmonella* vector could lead to the identification of promising drug discovery targets against this chronic, recalcitrant inflammatory skin disease.

## Supplementary Materials

The following are available online at https://www.mdpi.com/article/10.3390/microorganisms9081715/s1, Figure S1: Cytotoxicity of genetically engineered *S. typhimurium*, Figure S2: Relative green fluorescence was detected in the RAW 264.7 cells infected with either ST-miRTLR8 or ST-miR*CV*, Figure S3: The assessment of genetically engineered *S. typhimurium* accumulation in normal tissue, Figure S4: Total histology score plotted per group.
