# Supplementary material for: Bacterially Delivered miRNA-Mediated Toll-like Receptor 8 Gene Silencing for Combined Therapy in a Murine Model of Atopic Dermatitis: Therapeutic Effect of miRTLR8 in AD
Source: Microorganisms. 2021 Aug 12;9(8):1715. doi: 10.3390/microorganisms9081715 (PMC8401271; doi:10.3390/microorganisms9081715)
Supplement: Supplementary file 1 [file microorganisms-09-01715-s001.zip › microorganisms-1269333-supplementary.pdf]

Supplementary Figure S1.

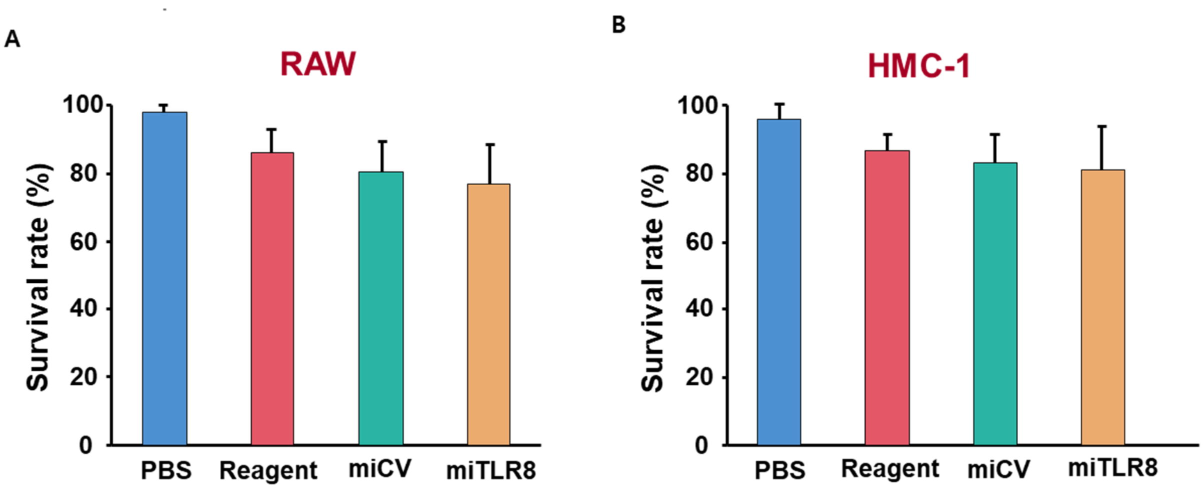

Figure S1. Cytotoxicity of genetically engineered *S.* Typhimurium. More than 80% of cell viability has been shown in RAW mouse macrophages (A) and HMC-1 human mast cells (B).

Supplementary Figure S2.

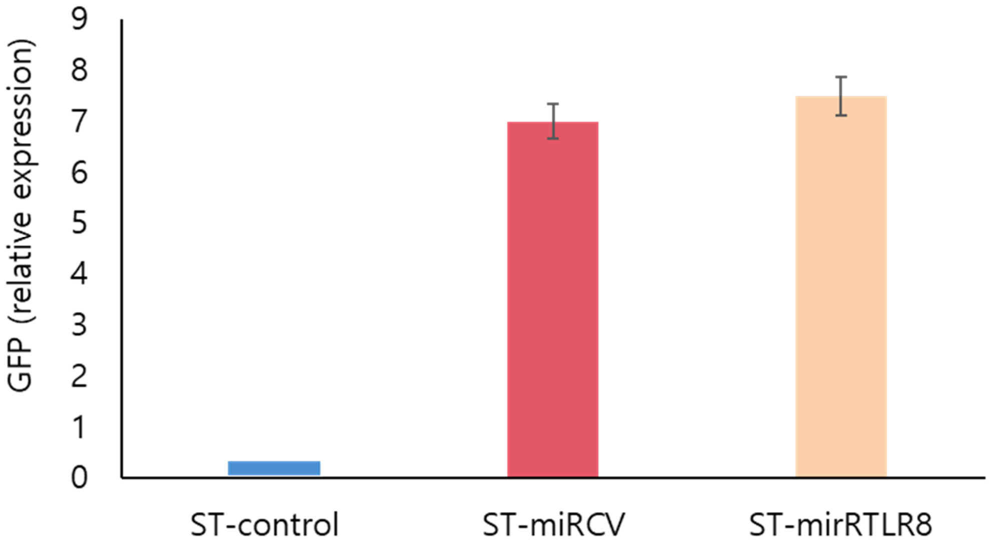

Supplementary Figure S2. Relative green fluorescence was detected in the RAW 264.7 cells infected with either ST-miRTLR8 or ST-miR*CV*.

Supplementary Figure S3.

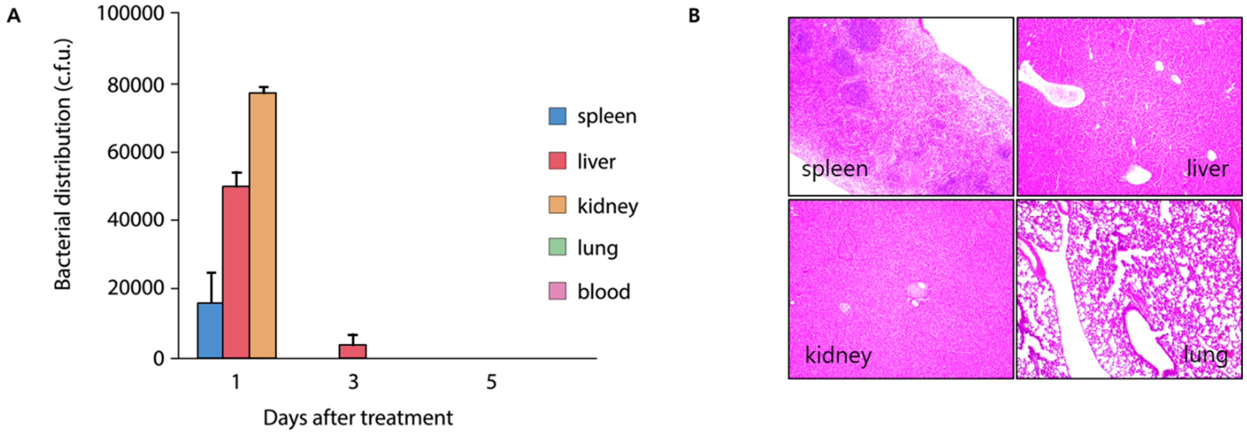

Figure S3. The assessment of genetically engineered *S. typhimurium* accumulation in normal tissue, ICR mice (n=5) were orally inoculated with 1.6x10^{8} colony forming units (CFU) of *S. typhimurium* for 3 days. (A) The lung, spleen, liver, kidney and blood from mice were lysed with 1% Triton X-100 for 5 minutes and then plated to determine the CFU counts. (B) Hematoxylin and eosin (HE) staining revealed no significant pathological changes in the lung, spleen, liver and kidney after treatment with the indicated *S.* Typhimurium.

Supplementary Figure S4.

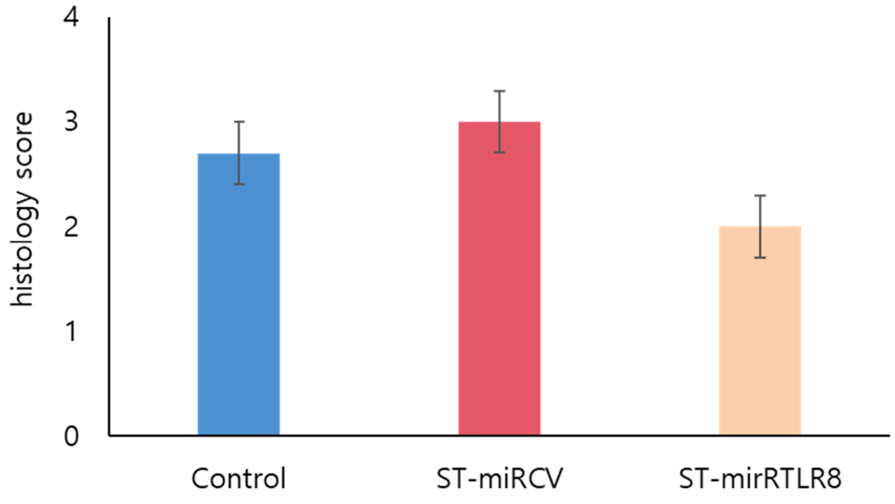

Figure S4. Total histology score plotted per group. Representative histological sections of skin were slightly decreased in the ST-miRTLR8-treated mice compared to the PBS or ST-miRCV groups.
